# Perinatal Mental Health Detection and Prediction Using Mobile Sensing Data: Systematic Review

**DOI:** 10.2196/101677

**Published:** 2026-09-25

**Authors:** Yifan Sun, Kemeng Che, Tella Lantta, Anna Axelin, Iman Azimi, Pasi Liljeberg

**Affiliations:** 1 Department of Computing, University of Turku, Yliopistonmaki, Turku, Southwest Finland, FI-20014, Finland, 358 0449508020; 2 Department of Nursing Science, University of Turku, Turku, Southwest Finland, Finland

**Keywords:** perinatal mental health, postpartum depression, wearable devices, smartphone sensing, digital phenotyping, machine learning, systematic review

## Abstract

**Background:**

Perinatal mental health disorders affect approximately 20% of pregnant and postpartum individuals, and are associated with substantial maternal and infant morbidity. Traditional assessment relies on infrequent, subjective self-reports. Mobile devices, including smartphones and wearables, offer opportunities for continuous and objective measurement, but evidence on their assessment utility in perinatal populations remains fragmented.

**Objective:**

This review aimed to examine the application of wearable devices and smartphones for detecting and predicting perinatal mental health outcomes, with emphasis on predictive performance, informative features, and methodological rigor.

**Methods:**

We conducted a systematic review following PRISMA (Preferred Reporting Items for Systematic Reviews and Meta-Analyses) guidelines (PROSPERO CRD420251249218). Six databases (PubMed, Web of Science, Scopus, PsycINFO, IEEE Xplore, and ACM Digital Library) were searched initially in January 2026 and supplemented by an amended search in June 2026, with no publication date restrictions. Evidence was synthesized narratively, and the risk of bias was assessed using PROBAST+AI (Prediction Model Risk of Bias Assessment Tool With AI extension).

**Results:**

The initial and supplementary searches yielded 1952 unique records after deduplication, of which 10 studies met the inclusion criteria. The included studies covered postpartum depression, prenatal stress, discrete emotions during pregnancy (eg, happiness, anxiety, and sadness), and maternal loneliness. High discrimination metrics were reported for postpartum depression in individual studies, including a multiclass area under the curve of 0.85, a binary area under the curve of 0.871, and an *F*_1_-score of 0.9872. Heart rate variability, GPS-derived mobility, physical activity, and sleep features were most frequently reported as useful, and their interpretation requires perinatal-specific contextualization. Methodological quality was limited, with 80% (12/15) of PROBAST+AI assessment units rated as having high overall quality concern or risk of bias, mainly due to small samples, limited validation, inadequate handling of missing data, and potential overfitting in the analysis domain.

**Conclusions:**

Mobile sensing shows preliminary potential for perinatal mental health assessment, but current evidence does not yet support clinical screening or decision-making, and independent external validation in perinatal populations is currently lacking. Progress toward clinical utility requires broader mental health outcome coverage, larger longitudinal cohorts, standardized analytical and reporting practices, adoption of modeling approaches better suited to perinatal trajectories, independent external validation, and human-centered monitoring designs.

## Introduction

Perinatal mental health issues are among the most common complications of childbearing, affecting approximately 20% of pregnant and postpartum individuals [1,2]. Among these conditions, peripartum depression is one of the most prevalent, with an estimated overall prevalence of 11.9%, but remains underdiagnosed and undertreated [3]. Without timely treatment, these conditions can result in substantial functional impairment, including diminished quality of life, difficulties in sustaining employment and social relationships, heightened risk of chronic psychiatric illness, and increased suicidality [1,3,4]. Beyond their direct impact on maternal well-being, these disorders can impair the quality of the mother-infant relationship, including disrupted bonding and less sensitive caregiving, and are associated with adverse outcomes in offspring’s cognitive, emotional, and behavioral development [5]. Recognizing these impacts, the World Health Organization (WHO) identifies perinatal mental health as a major public health challenge and highlights the need for early identification and management [6].

Traditional mental health assessments typically rely on clinical interviews and self-report scales, which are often subjective and infrequently administered [7], limiting their capacity for daily monitoring and early identification. The widespread adoption of smartphones and wearable devices has created new opportunities for continuous and objective mental health assessment. Smartphones are well suited for capturing behavioral and contextual data due to their pervasive integration into daily life. Passive smartphone sensing enables the collection of information on screen use, app engagement, communication activity, and GPS-derived mobility patterns. These data can be further transformed into higher-level behavioral indicators, such as daily routines, social interaction patterns, and physical activity regularity, which have been associated with mental health outcomes [7].

Wearable devices complement smartphone sensing by enabling continuous monitoring of physiological and physical signals directly from the body. Built-in sensors, such as accelerometers, provide measures of movement intensity, sleep, and energy expenditure [8]. In addition, many wearables integrate specialized sensors to capture peripheral physiological signals, including photoplethysmography (PPG) and electrodermal activity [9]. These signals reflect autonomic nervous system (ANS) activity, which is increasingly recognized as a core effector system linking stress, emotion, and physiological processes in psychophysiological research [10].

Building on these capabilities, smartphones and wearable devices have been increasingly investigated for the detection and prediction of mental health conditions, including depression [11], anxiety [12], and bipolar disorder [13]. In line with these developments, there is growing interest in applying these approaches to perinatal mental health research [14], where continuous, real-world monitoring may be particularly advantageous.

However, the current evidence base for predicting perinatal mental health outcomes using wearable and smartphone data remains limited and inconclusive. Existing evidence on digital mental health monitoring has been established primarily in general populations and may not generalize to perinatal individuals, as the physiological and behavioral features used to infer mental states undergo substantial normative changes during this period. For example, ANS activity indexed through heart rate variability (HRV) shifts markedly across gestation [15-17], overlapping with patterns typically associated with depression [18], thereby complicating interpretation. Sleep disruption, reduced physical activity, and increased nighttime phone use may also reflect normative pregnancy- and caregiving-related changes rather than psychopathology [19-22]. Furthermore, studies in perinatal populations show considerable variation in sensing devices, derived data, and analytical approaches [22-24]. Such heterogeneity, along with potential methodological limitations, hinders clear conclusions regarding effectiveness, the most relevant sensing features, and methodological robustness.

One previous review by Novick et al [14] surveyed technology-based approaches for supporting perinatal mental health, providing a broad overview of available technologies, their applications to perinatal mood and anxiety disorders, and the supporting evidence. However, its scope was not specifically focused on the use of wearable devices and smartphones, nor on the monitoring or prediction of mental health outcomes. Furthermore, it did not use a systematic approach to evidence synthesis and offered limited technical detail. A focused systematic review is therefore needed to synthesize the current evidence and identify key gaps.

To address these gaps, this review provides a focused, systematic synthesis of current evidence on the use of wearable devices and smartphones for detecting and predicting perinatal mental health outcomes. Specifically, we sought to answer the following questions:

1. Can wearables and smartphones effectively detect or predict perinatal mental health outcomes?
2. What sensing modalities and derived features have been used, and which have been reported as useful in individual studies?
3. What analytical approaches have been applied, and what are their performance outcomes and methodological limitations?

## Methods

This review was completed in accordance with the PRISMA (Preferred Reporting Items for Systematic Reviews and Meta-Analyses) guidelines (Checklist 1) [25]. The review protocol was preregistered with the International Prospective Register of Systematic Reviews (PROSPERO) with registration number CRD420251249218.

### Search Strategy

We conducted the database search in 2 stages: an initial search and a supplementary search.

The initial search was conducted in January 2026 in PubMed, Web of Science, Scopus, PsycINFO, IEEE Xplore, and ACM Digital Library, with no publication date restrictions. The search strategy was organized around 4 conceptual domains: population, mental health outcomes, mobile sensing technologies, and analytical approaches. Within each domain, related keywords and synonyms were combined using the Boolean operator “OR,” and the 4 domains were combined using “AND.” The search strategy was developed by the review team and refined through preliminary searches in PubMed. It was not formally peer reviewed.

The initial search strings did not explicitly include schizophrenia, bipolar disorder, or eating disorders, although these conditions were specified in the registered protocol. Following peer review, a supplementary search was conducted in June 2026 as an amendment to the protocol. Condition-specific terms for schizophrenia, bipolar disorder, and eating disorders were added to the initial search strings across all databases. In addition, the ACM Digital Library search was expanded from the abstract field alone to both the title and abstract fields. Records retrieved through the supplementary search were imported into Covidence (Veritas Health Innovation), deduplicated within the supplementary search and against the initial search records, and screened using the same eligibility criteria and procedures.

**Figure 1.**
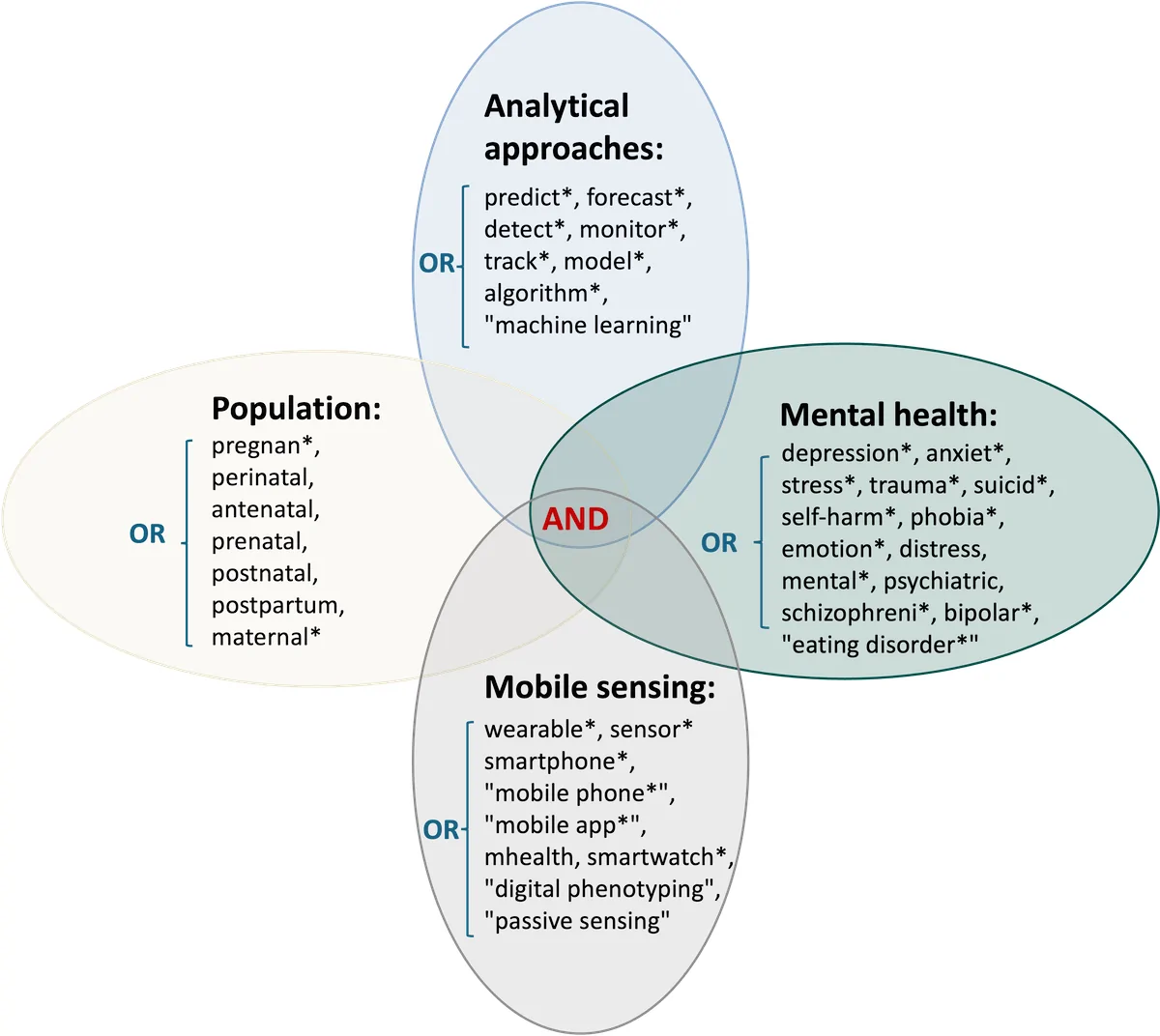
Conceptual structure and keywords of the search strategy.

Figure 1 illustrates the conceptual structure and keywords used in the supplementary search. The complete initial and supplementary search strategies are provided in Multimedia Appendix 1.

### Inclusion and Exclusion Criteria

Eligibility criteria were defined using the SPIDER (sample, phenomenon of interest, design, evaluation, and research type) framework [26] instead of the more commonly used PICO (patient, intervention, comparison, and outcome) framework [27]. As PICO is oriented toward population, intervention, comparator, and outcome, it was less suited to this review, in which the included studies were primarily observational or predictive and did not involve intervention-comparator contrasts. SPIDER was considered more aligned with our aim of synthesizing analytical and predictive evidence.

Eligible studies were required to include individuals during pregnancy or up to 1 year postpartum; use passively sensed data from wearable devices or smartphones as model inputs, either alone or in combination with other types of data; and apply statistical or computational methods to detect or predict mental health outcomes. Mental health outcomes were required to be assessed using self-report instruments or clinical diagnoses and to serve as reference standards for evaluating model performance. As preliminary searches indicated that evidence in this area was sparse, we also included studies using nonvalidated self-report items. Eligible publications were required to be quantitative empirical studies published in English as peer-reviewed journal articles or full-text conference papers. The detailed eligibility criteria, organized by SPIDER element, are presented in Table 1.

**Table 1.** Eligibility criteria.

SPIDER^a^ element	Inclusion criteria	Exclusion criteria
Sample (S)	Studies involving individuals during pregnancy and up to 1 year following childbirth [28].	Studies in which perinatal data cannot be separated from nonperinatal populations
Phenomenon of interest (PI)	Studies using data collected via wearable devices or smartphones to detect or predict mental health conditions were eligible. The data of interest are passively sensed data obtained from wearable devices or smartphones, including physical activity, sleep patterns, physiological biosignals (eg, heart rate), and device-derived behavioral or usage data. The mental health problems of interest include depression, anxiety, stress, trauma-related symptoms, self-harm, bipolar disorder, mood disorder, eating disorder, and schizophrenia.	Not applicable
Design (D)	Passively sensed data are used as inputs in statistical or computational modeling methods to estimate, classify, or forecast mental health outcomes.	Not applicable
Evaluation (E)	Mental health outcomes are assessed using self-report instruments or clinical diagnoses and serve as reference standards for evaluating predictive performance. Predictive performance is evaluated using metrics appropriate to the analytical approach (eg, correlation coefficients, *P* values, accuracy, and mean absolute error).	Not applicable
Research type (R)	Quantitative empirical studies only.	Not applicable
Publication characteristics	Peer-reviewed journal articles and full-text conference papers published in English.	Not applicable

a SPIDER: sample, phenomenon of interest, design, evaluation, and research type*.*

### Study Selection

Study selection was performed using Covidence [29], a web-based collaboration software platform that streamlines the production of systematic and other literature reviews. Three independent reviewers (YS, KC, and TL) participated in the study selection process. After duplicate removal, 2 reviewers (YS and KC) independently screened all titles and abstracts, followed by independent full-text assessment of potentially eligible records. The reasons for exclusion were recorded at the full-text stage. Disagreements were resolved by the third reviewer (TL).

### Data Extraction and Synthesis

Data were extracted using a predefined data extraction form developed by the review team and managed in Microsoft Excel. The predefined data extraction form included information on study and participant characteristics (author, year, population stage, sample size, and study duration), mental health outcomes (targeted outcome and the corresponding outcome measures), digital sensing setup and data collected (mobile device or platform used and the data collected), and modeling approaches (the analytical goal, the inputs and outputs used for analysis, the analysis strategy, the evaluation metrics, and the key findings).

During data extraction, we classified each data source into 1 of 4 categories because participant burden, missingness mechanisms, privacy implications, and clinical scalability may differ substantially by data type. Passive sensing data refer to data acquired automatically from device sensors without deliberate participant input, such as HRV features and device usage logs. Active self-report data refer to data requiring deliberate participant input, such as daily diaries and ecological momentary assessment (EMA) prompts. Clinical data refer to information obtained from medical records or clinician assessments. Other app-derived data refer to data generated through app engagement that were neither purely passive nor conventional self-report, such as voice diary acoustic features.

To ensure consistency in data extraction, a second reviewer (KC) independently checked a randomly selected subset of 4 (40%) of the 10 included studies. This subset served as a calibration sample for the extraction process, consistent with Cochrane guidance recommending that data collection forms be piloted within the review team to improve consistency [30]. No substantive discrepancies were identified between reviewers; the few differences concerned the wording of extracted entries rather than their content and were resolved through discussion. As this calibration process did not identify substantive inconsistencies, the remaining studies were extracted by the primary reviewer (YS) using the agreed extraction framework.

### Quality Assessment

The methodological quality and risk of bias of included studies were assessed using PROBAST+AI (Prediction Model Risk of Bias Assessment Tool With AI extension) [31], an updated version of PROBAST [32] designed to evaluate prediction models developed using either traditional statistical methods or AI techniques.

The tool consists of 2 components: model development, which assesses methodological quality, and model evaluation, which assesses the risk of bias in estimated predictive performance. Both components are appraised using separate signaling questions and cover 4 domains: participants and data sources, predictors, outcomes, and analysis. Applicability is additionally assessed for participants and data sources, predictors, and outcomes.

We applied the tool following a 3-step process. First, each prediction model was classified as model development only, model evaluation only, or both. Second, signaling questions within each domain were answered as yes, probably yes, probably no, no, no information, or not applicable; responses were then used to assign domain-level ratings of low, high, or unclear. Third, domain ratings were combined into an overall judgment for each component. Low concern or low risk of bias required all applicable domains to be rated low (ie, high quality). High concern or high risk of bias was assigned when at least one applicable domain was rated high (ie, low quality). Unclear was assigned when at least one applicable domain was rated unclear and none was rated high.

Conference papers were appraised using the same signaling questions and procedures as peer-reviewed journal articles. When the available information was insufficient to answer a signaling question, the item was rated as unclear.

In this review, the assessments were conducted independently by 2 reviewers (YS and KC), with disagreements resolved through discussion and, when needed, consultation with a third reviewer (AA). The PROBAST+AI results were used to characterize the methodological quality and risk of bias of the included studies and to inform the cautious interpretation of their findings. They were not used to exclude or weight studies; all studies contributed equally to the narrative synthesis.

## Results

### Search Results and Study Selection

The initial database search identified 3741 records. After deduplication, 1636 records remained for title and abstract screening. The supplementary search identified 4456 records. After removing duplicates within the supplementary search and against the initial search records, 316 new records remained for screening.

**Figure 2.**
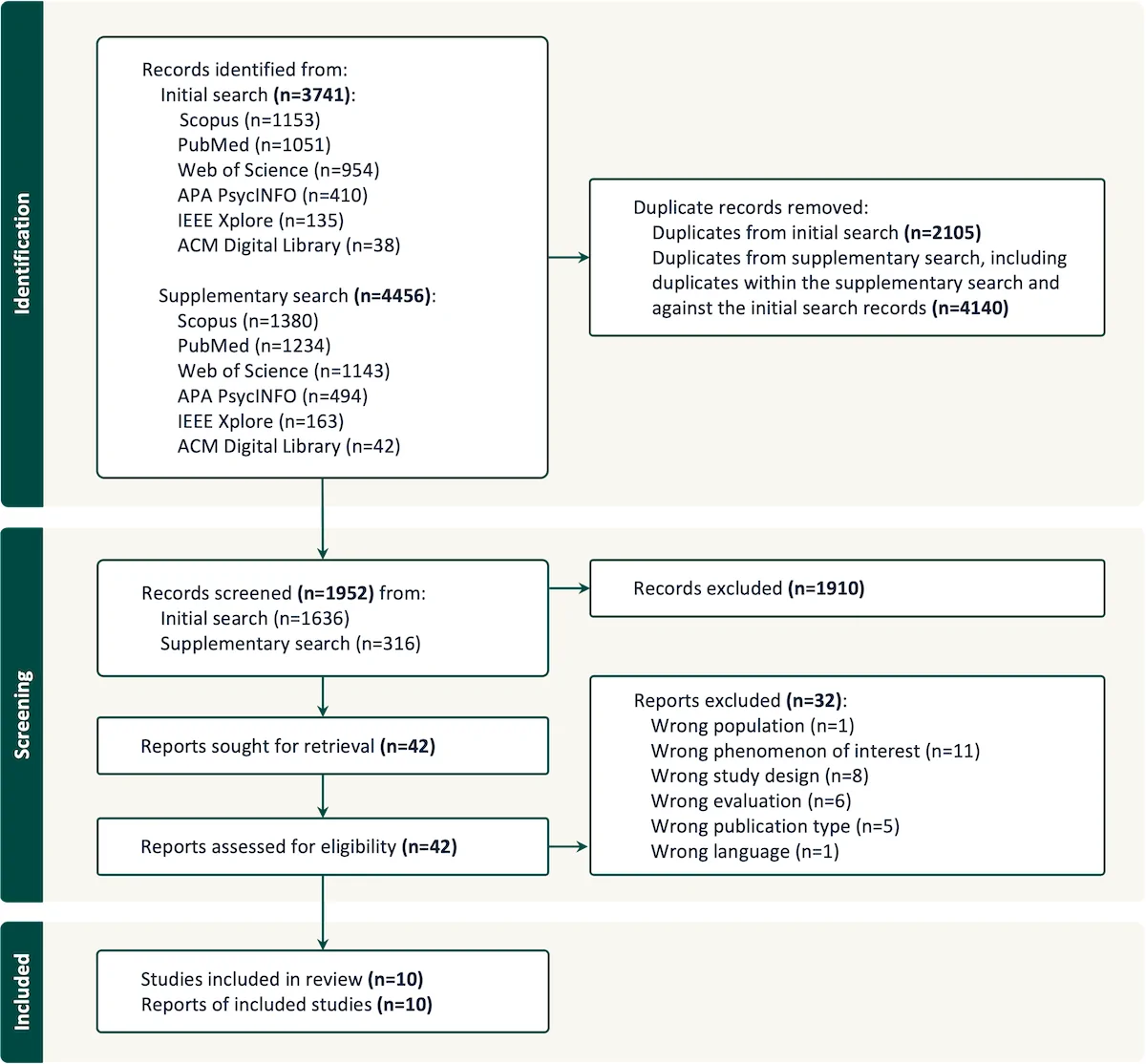
PRISMA (Preferred Reporting Items for Systematic Reviews and Meta-Analyses) flowchart.

Across the initial and supplementary searches, 1952 records underwent title and abstract screening, of which 1910 were excluded. The remaining 42 reports were sought for retrieval and assessed for eligibility. Of these, 32 reports were excluded with documented reasons, and 10 studies, represented by 10 reports, were included in the final review. The PRISMA flow diagram (Figure 2) summarizes the study selection process.

### Overview of Study Characteristics

The included studies were conducted across North America, Europe, and Asia. Five studies were from the United States [23,33-36], 2 from Europe, namely Finland and Italy [37,38], and 3 from Asia, namely Japan and China [22,24,39]. Full details of each study are provided in Table 2.

**Table 2.** Characteristics, mental health outcomes, and mobile sensing setup of the included studies.

Study, year	Population stage	Sample size	Duration	Target outcome	Outcome measures	Mobile device or platform	Data collected
Faherty et al, 2017 [33]	Pregnancy (<32 weeks’ gestation at enrollment) at risk for depression (PHQ-9^a^ ≥5)	36	8 weeks	Perinatal depression, daily mood, and anxiety	PHQ-2^b^ (daily); PHQ-9 or GAD-7^c^ (biweekly)	Smartphone app—Ginger.io	Passive sensing data: (1) mobility (daily travel distance, maximum travel radius); and (2) phone usage (number and duration of phone calls and text messages)
Hurwitz et al, 2024 [34]	Across prepregnancy (up to 2 years before pregnancy), pregnancy, and postpartum	<59^d^	Not reported	PPD^e^	Medical records (PPD diagnosis or antidepressant exposure during postpartum)	Wearable—Fitbit	Passive sensing data: (1) HR^f^ and HRV^g^ features (average HR, HR SD, minimum HR, Q1 HR, median HR, Q3 HR, and maximum HR); and (2) physical activity (step count, activity calories, calories burned during the basal metabolic rate, calories out, fairly active minutes, lightly active minutes, marginal calories, sedentary minutes, and very active minutes)
Jose et al, 2025 [23]	Postpartum	1503	Not reported	PPD	Self-report on psychological health, perceived stress, and lifestyle (no validated scales or thresholds reported)	Not reported	Passive sensing data: HR and HRV features, sleep quality, and physical activity Other app-derived data: social interaction logs (daily records of social encounters; collection method not reported) Clinical data: prenatal, delivery, and immediate postnatal examination records
King et al, 2019 [36]	Pregnancy (first or second trimester at enrollment)	18^h^	2 days	Stress	EMA^i^: 12 items via text messages (3‐9 times over 2 days), including PSS-4^j^, BinaryStress, LikertStress, Worried, Sad, Angry, Content, Happy, and Excited	Wearable—BioStampRC (MC10)	Passive sensing data: HRV and IBI^k^-derived statistical features (0.1‐0.2 Hz LF^l^, 0.2‐0.3 Hz MF^m^, 0.3‐0.4 Hz HF^n^, LF/HF^o^, variance, quartile deviation, RMSSD^p^, SDSD^q^, pNN20^r^, pNN50^s^, mean, median, mode, minimum, maximum, range, root-mean-square, zero crossing, kurtosis, skew, IQR percentiles, count<mean, count>mean)
Li et al, 2025 [24]	Postpartum (≤6 weeks postpartum at enrollment)	102^t^	12 weeks	PPD	EPDS^u^ and clinical diagnosis using *DSM-5*^v^	Smartphone app—Maternal Love Guardian (version 1.2.4)	Passive sensing data: (1) phone usage (frequency, duration, and app types); and (2) physical activity and mobility (GPS-derived activity range) Other app-derived data: voice features (speaking rate, pitch, and pauses from weekly voice diaries). Active self-report data: (1) weekly self-reported scales (EPDS, PSSS^w^, WCQ-P^x^, PSQI^y^); (2) daily diaries (mood, sleep, breastfeeding, infant interaction); and (3) user feedback
Li et al, 2022 [39]	Pregnancy (23‐32 weeks’ gestation at enrollment)	53	1 week	Emotional states (happy, anxious, sad, and frustrated)	Self-reported emotion logging (smartphone app, icon selection; no validated scales or thresholds reported)	Wearable—MyBeat (Union Tool Co)	Passive sensing data: HRV features (CVRR^z^, SDNN^aa^, RMSSD, NN50^ab^, pNN50, LF, HF, LF/HF)
Ng et al, 2022 [35]	Pregnancy (10‐18 weeks’ gestation at enrollment)	16	12 weeks	Stress	EMA: 12 items via text messages (5 times per day), including PSS-4, BinaryStress, LikertStress, Worried, Sad, Angry, Content, Happy, and Excited	Wearable—BioStampRC (MC10)	Passive sensing data: (1) HRV and IBI-derived statistical features (RMSSD, SDSD, pNN20, pNN50, NN20^ac^, NN50, LF, MF, HF, LF/HF, mean, median, mode, count, count<mean, count>mean, SD, covariance, minimum, maximum, range, root-mean-square, kurtosis, skewness, IQR, zero crossing, and percentiles); (2) HRV-derived duration-based features (17 features capturing time spent physiologically stressed while wearing the sensor, eg, Total_Stress)
Sarhaddi et al, 2023 [37]	Pregnancy and postpartum (12‐15 weeks’ gestation at enrollment)	31	9‐10 months	Maternal social loneliness	UCLA^ad^ Loneliness Scale (collected at gestational week 36 and 12 weeks postpartum)	Wearable—Samsung Gear Sport smartwatch; smartphone app (custom cross-platform)	Passive sensing data: (1) HR and HRV features (AVNN^ae^, RMSSD, SDNN, LF, HF, and LF/HF ratio); (2) sleep features (total sleep time [TST], wake after sleep onset [WASO], average hand movement, sleep quality indicator [WASO ≤20 min], and sufficient sleep indicator [TST 7‐8.5 h]); (3) physical activity features (step count, walking steps, running steps, distance, activity duration, activity intensity, sedentary time [awake time without walking or running], and sufficient activity indicator [daily steps >7000])
Singh Solorzano et al, 2022 [38]	Pregnancy (second or third trimester at enrollment)	135	1‐6 months, with assessments at prepartum and 1 month postpartum	PPD	EPDS	Smartphone app—HRV Camera (ECG^af^ for Everybody)	Passive sensing data: HRV feature (RMSSD)
Tang et al, 2025 [22]	Postpartum (≤1)	21	4 weeks	PPD	EPDS (weekly)	Smartphone app—ZeroPPD (iOS)	Passive sensing data: (1) GPS-derived mobility features (number of significant locations, time spent at these locations, location entropy, radius of gyration, and temporal regularity in movement [eg, circadian rhythms]); (2) phone usage features (unlock frequency, unlocked session duration, first interaction time, and last interaction time); and (3) physical activity features (stationary bout count and duration, and active bout count and duration [eg, walking, running, cycling, and automotive]) Active self-report data: weekly summaries of baby crying and maternal awakenings

a PHQ-9: Patient Health Questionnaire-9.

b PHQ-2: Patient Health Questionnaire-2.

c GAD-7: Generalized Anxiety Disorder-7.

d PPD: n<20; non-PPD: n=39; modeling analysis used PPD data only.

e PPD: postpartum depression.

f HR: heart rate.

g HRV: heart rate variability.

h Eighteen pregnant women were recruited, and 17 were used in the analysis.

i EMA: ecological momentary assessment.

j PSS-4: 4-item Perceived Stress Scale.

k IBI: interbeat interval.

l LF: power of the low-frequency band.

m MF: power of the medium-frequency band.

n HF: power of the high-frequency band.

o LF/HF: ratio of LF-to-HF power.

p RMSSD: root-mean-square of successive differences between RR intervals.

q SDSD: SD of successive RR interval differences.

r pNN20: percentage of successive RR intervals that differ by more than 20 ms.

s pNN50: percentage of successive RR intervals that differ by more than 50 ms.

t Intervention: n=54; control: n=48; analysis used intervention data only.

u EPDS: Edinburgh Postnatal Depression Scale.

v *DSM-5*: *Diagnostic and Statistical Manual of Mental Disorders* (Fifth Edition).

w PSSS: Perceived Social Support Scale.

x WCQ-P: Ways of Coping Questionnaire (revised).

y PSQI: Pittsburgh Sleep Quality Index.

z CVRR: coefficient of variation of RR intervals.

aa SDNN: SD of NN intervals.

ab NN50: successive RR intervals that differ by more than 50 ms.

ac NN20: successive RR intervals that differ by more than 20 ms.

ad UCLA: University of California, Los Angeles.

ae AVNN: average of NN intervals.

af ECG: electrocardiogram.

The studies covered different stages of the perinatal period. Five studies focused on pregnancy, with participants typically monitored between 10 and 32 weeks’ gestation [33,35,36,38,39]. Three studies examined the postpartum period, following mothers from childbirth up to 1 year postpartum [22-24]. The remaining 2 studies adopted a longitudinal design spanning multiple stages, from prepregnancy or early pregnancy through the postpartum period [34,37].

Data collection duration varied substantially, ranging from short-term intensive monitoring over 2 days to 1 week [36,39], to follow-up periods of 4 to 12 weeks [22,24,33,35]. Longer studies extended monitoring to 9 to 10 months [37] or collected repeated measurements across multiple perinatal stages [34,38]. Two studies did not clearly report their monitoring duration [23,34].

Sample sizes also varied widely, from small exploratory or pilot cohorts of 16 to 40 participants [22,33,35-37], to mid-sized samples of 50 to 135 participants [24,34,38,39], and 1 large-scale dataset including 1503 participants [23].

### Mental Health Outcomes Assessed

The primary outcome of interest across the included studies was postpartum depression (PPD) or perinatal depression, with most studies focusing on identification [22,24,33,34], prediction [38], and risk stratification [23]. In addition to PPD, studies also examined maternal stress [35,36], perinatal anxiety [33], maternal social loneliness [37], and specific perinatal emotional states (eg, happiness, anxiety, sadness, and frustration) [39].

The measures used to assess these outcomes involved a combination of validated clinical scales and innovative digital assessment tools. The Edinburgh Postnatal Depression Scale (EPDS) [40] was the most frequently used tool for screening PPD and assessing symptom severity [22,24,38]. Other validated instruments included the Patient Health Questionnaire (PHQ)-9 and PHQ-2 [41] for depressive symptoms [33]. Perceived stress was commonly measured using the 4-item Perceived Stress Scale [35,36,42]. Anxiety was specifically assessed via the Generalized Anxiety Disorder-7 [33,43]. Furthermore, micro-EMAs were used to capture real-time reports to reduce participant burden [35,36]. The revised University of California, Los Angeles Loneliness Scale (12-item version) [44] was used to detect social and emotional loneliness [37].

### Devices, Sensing Modalities, and Features

Four studies primarily used smartphone-based platforms, including passive behavioral sensing apps and 1 smartphone camera–based PPG tool. Faherty et al [33] used the Ginger.io platform on Android devices to passively capture GPS-derived mobility (total distance traveled on foot) and radius of travel, alongside EMA prompts for daily and weekly mood. Tang et al [22] deployed “ZeroPPD,” an iOS app built on the AWARE framework [45], collecting data, including step counts, GPS location, and screen state, from which they extracted more than 700 raw features, subsequently reduced to approximately 70 using low-variance filtering and regularization. Li et al [24] used the “Maternal Love Guardian” app to capture behavioral, emotional, cognitive, and voice data, enabling the derivation of a digital phenotyping risk score. Singh Solorzano et al [38] used the PPG smartphone app, “HRV Camera” [46], to capture cardiac interbeat intervals via the device camera, validating this approach against concurrent laboratory electrocardiogram (ECG) in a preliminary substudy.

Five studies used wearable sensors capable of capturing continuous physiological signals. Hurwitz et al [34] used a large secondary dataset based on data from consumer-grade Fitbit devices to derive physiological and behavioral features, including heart rate (HR) features (eg, average and minimum HR) and physical activity–related features (eg, sum steps and activity calories). King et al [36] used the BioStampRC [47] flexible ECG patch, worn for approximately 12 hours in naturalistic settings, to extract HRV features, such as root-mean-square of successive differences (RMSSD) between RR intervals and SD of successive RR interval differences. Li et al [39] used the MyBeat [48] wearable chest sensor to record HRV features during free-living conditions, including the coefficient of variation of RR intervals (CVRR), SD of NN intervals (SDNN), RMSSD, absolute power of the high-frequency (HF) band, and absolute power of the low-frequency (LF) band. Ng et al [35] similarly used the BioStampRC ECG sensor over 12 weeks, collecting 4157 hours of data to derive HRV features and HRV-derived duration-based features. Sarhaddi et al [37] leveraged a Samsung Gear Sport smartwatch [49] to continuously capture HRV features, sleep parameters, and physical activity.

Jose et al [23] used a more comprehensive multimodal data collection strategy, integrating e-survey questionnaires, wearable sensor data (HRV, sleep quality, and physical activity), social interaction logs, and clinical records from 1503 participants. However, the paper did not specify the wearable device or platform used or how the social interaction logs were collected, leaving these key details unclear.

### Analytical Approaches and Findings

#### Overview

The findings of the included studies are summarized across 4 domains of perinatal mental health: perinatal depression, prenatal stress, emotional states during pregnancy, and maternal loneliness. These findings are presented alongside the analytical approaches used, which ranged from traditional inferential statistical models to supervised machine learning and meta-learning frameworks. The analytical approaches, evaluation metrics, and key findings of the included studies are summarized in Table 3. To support the interpretation of the reported performance metrics, this section also summarizes the model validation methods used in the end.

**Table 3.** Analytical approaches and findings.

Study	Goal	Data for analysis	Analysis strategy	Evaluation metrics	Findings
Faherty et al, 2017 [33]	Analyze associations between (1) self-reported mood and movement patterns and (2) PHQ-9^a^/GAD-7^b^ scores and movement patterns	Inputs: passive sensing data (mobility and phone usage features) Outputs: daily mood, PHQ-9 scores, and GAD-7 scores	Statistical analysis model: (1) generalized linear mixed effects models to assess the association between mood and movement patterns; and (2) generalized linear mixed effects models compared movement patterns between 2 depression groups.	β estimate (95% CI), ratio of geometric means (95% CI), and *P* value	Daily mood was not associated with same-day travel distance or travel radius; however, changes in mood from the previous day were significantly associated with travel radius, with worsened mood linked to reduced radius (*P*=.03). Women with milder depression symptoms had a larger daily travel radius (2.7 miles) than those with more severe symptoms (*P*=.04), with no difference in travel distance. No significant relationships between anxiety and either daily travel distance or radius.
Hurwitz et al, 2024 [34]	Classify 4 perinatal periods, including prepregnancy, pregnancy, postpartum, and PPD	Inputs: passive sensing data (HR^c^, HRV^d^, and physical activity features) Outputs: 4 perinatal periods	Individualized multinomial machine learning classification model: (1) candidate models: RF^e^, GLM^f^, SVM^g^, and KNN^h^; (2) optimization or evaluation: 3 repeats of 10-fold cross-validation with a tuning length of 5; and (3) explainability: RF feature importance, SHAP^i^, and permutation-based methods.	Precision, recall, *F*_1_-score, sensitivity, specificity, mAUC^j^, and κ statistic	RF performed best (mAUC=0.85; κ=0.80), followed by GLM (mAUC=0.82; κ=0.74), SVM (mAUC=0.75; κ=0.72), and KNN (mAUC=0.74; κ=0.62). Key predictors included calories BMR^k^, average HR, quartile 1 HR, lightly active minutes, and minimum HR.
Jose et al, 2025 [23]	Classify PPD^l^ risk levels (no classification criteria or thresholds reported)	Inputs: passive sensing data (HR, HRV, sleep quality, and physical activity features); other app-derived data (social interaction logs); clinical data (prenatal, delivery, postnatal examination records) Outputs: PPD risk levels	Meta-learning framework (CAML^m^): (1) component models within CAML: LSTMs^n^ for temporal analysis of time series data from wearables and social interactions; CNNs^o^ for processing lifestyle images or heatmaps containing risk information of PPD; XGBoost^p^ or gradient boosting for ranking primary risk factors; (2) evaluation: k-fold cross-validation; and (3) explainability: SHAP and LIME^q^.	Accuracy, precision, recall, and *F*_1_-score	The CAML framework achieved accuracy=0.987, precision=0.986, recall=0.9885, *F*_1_-score=0.9872 in predicting PPD risk and outperformed baseline models, including gradient boosting, LSTM, XGBoost, CNN, and random forest.
King et al, 2019 [36]	Validate a model for binary stress classification (stress vs nonstress)	Inputs: passive sensing data (HRV and IBI^r^-derived statistical features) Outputs: 2 stress levels (using different stress labels)	Machine learning classification model: (1) candidate models: SVM; and (2) optimization or evaluation: grid search; features were extracted in multiple time windows (1‐60 min), and model performance was evaluated across 3 outcome definitions (binary stress, Likert-scale stress, and “worried” stress) and different window lengths.	Accuracy	Specific accuracy was not reported. The relative ranking of labels varied by time window: in shorter windows, “worried” stress performed better, whereas in longer windows, Likert-scale and binary stress labels performed better.
Li et al, 2025 [24]	Classify PPD risk levels (low, moderate, and high)	Inputs: passive sensing data (phone usage, physical activity, and mobility); other app-derived data (voice features); active self-report data (scales and daily diaries) Outputs: 3 PPD risk levels	Statistical analysis model: (1) linear weight to calculate baseline clinical risk scores using self-reported scales data; (2) multivariable logistic regression to calculate phenotypic risk scores using app data-derived features; and (3) linear weight to integrate clinical and phenotypic risk scores (6:4) into overall PPD risk levels (low, moderate, and high).	Accuracy, sensitivity, specificity, positive predictive value, negative predictive value, and AUC^s^	The model achieved optimal performance indicators at 3 weeks postpartum, with 90% sensitivity, 84.1% specificity, and AUC=0.871. Women with PPD showed significantly reduced sleep duration (6.1 vs 7 h) and efficiency (74.2% vs 83.8%), reduced physical activity (4389 vs 6542 steps per day), smaller social activity radius (2.3 vs 4.4 km), and slower speaking rate (148 vs 175 words per minute).
Li et al, 2022 [39]	Classify emotional states (happy, anxious, sad, and frustrated)	Inputs: passive sensing data (HRV features) Outputs: 4 emotional states	Machine learning classification models: (1) candidate models: KNN, SVM, LR^t^, NB^u^, SGD^v^, DT^w^, RF, GB^x^, XGBoost, and ANN^y^; (2) optimization: RandomizedSearchCV for hyperparameter search; (3) explainability: random forest feature importance.	Accuracy, precision, *F*_1_-score, sensitivity, specificity, and AUC	RF achieved the highest AUC=0.70, followed by GB (0.69), ANN (0.68), XGBoost (0.65), SVM/LR/DT (0.65), SGD (0.64), KNN (0.61), and NB (0.52). CVRR^z^ had the highest importance in emotion prediction, followed by RMSSD^aa^, SDNN^ab^, HF^ac^, LF^ad^/(LF+HF), pNN50^ae^, LF/HF, with NN50^af^ contributing the least.
Ng et al, 2022 [35]	Classify next-day physiological and perceived stress separately (stressed vs nonstressed)	Inputs: passive sensing data (HRV and HRV-derived duration-based features); active self-report data (EMA^ag^); intervention features (cognitive behavioral therapy–based intervention exposure) Outputs: 2 physiological stress levels, 2 perceived stress levels	Machine learning classification model: (1) candidate models: GB, SVM, adaptive boosting, NB, DT, and RF to predict next-day physiological and perceived stress using all features with 5-fold cross-validation; (2) optimization: correlation-based feature subset selection to select the optimal subset of features; Bayesian optimization to automatically tune hyperparameters; (3) explainability: SHAP.	Precision, recall, and *F*_1_-score	RF achieved the best performance for predicting next-day physiological stress (*F*_1_-score=0.84) and perceived stress (*F*_1_-score=0.74). Any subset of sensor, EMA, or intervention data achieved the reported performance for next-day physiological stress under internal cross-validation; EMA data were necessary for perceived stress prediction. Top 5 features for physiological stress prediction included consecutive stress minutes, intervention count, stress-minute percentage, number of children, and PSS overcome. Top 5 features for perceived stress prediction included PSS-4, PSS control, PSS overcome, number of children, happy stress, and content stress.
Sarhaddi et al, 2023 [37]	Classify maternal social loneliness levels (loneliness vs nonloneliness)	Inputs: passive sensing data (HRV, sleep, and physical activity features) Outputs: 2 loneliness levels	Machine learning classification models: Candidate models: DT and GB. Optimization or evaluation: recursive feature elimination to select the optimal subset of features; leave-one-participant-out cross-validation. Explainability: decision tree and gradient boosting feature importance.	Precision, recall, *F*_1_-score, weighted *F*_1_-score, sensitivity, specificity, and AUC	Gradient boosting and decision tree models predicted maternal social loneliness with weighted *F*_1_-scores of 0.897 and 0.872, respectively; gradient boosting showed higher specificity than decision tree. Loneliness was highly associated with lower daytime activity intensity and distribution, lower resting HR, and HRV. Top decision tree features were activity intensity and step-count kurtosis, followed by resting SDNN, LF, LF/HF, and activity duration (maximum, median, and mean). Top gradient boosting features were activity intensity and step-count kurtosis, followed by step and activity distribution metrics, sedentary time, and HRV features (resting HR, LF/HF, SDNN, and AVNN^ah^).
Singh Solorzano et al, 2022 [38]	Analyze prepartum HRV as a predictor of PPD	Inputs: passive sensing data (HRV feature) Outputs: EPDS^ai^ scores	Statistical analysis model: (1) Pearson correlation to examine associations between prepartum RMSSD and depressive symptoms at both prepartum and postpartum; (2) Hierarchical linear regression to assess whether prepartum RMSSD predicted postpartum depressive symptoms after adjusting for covariates (age, education, pregnancy trimester, prepartum BMI, and prepartum depressive symptoms).	Correlation coefficient (*r*), standardized regression coefficient (β), and *P* values	Lower prepartum RMSSD predicted greater postpartum depressive symptoms (β*=*−0.217; *P*=.01); hierarchical linear regression explained 30% of the variance in PPD. Prepartum depressive symptoms were associated with PPD (β=.447; *P*<.001).
Tang et al, 2025 [22]	Explore passive mobile sensing for assessing postpartum mental health and identify behavioral indicators associated with PPD symptoms	Inputs: passive sensing data (mobility, phone usage, physical activity features); active self-report data (baby crying and maternal awakenings) Outputs: EPDS scores	Statistical analysis model: (1) Low-variance filtering and regularization-based feature selection with internal cross-validation; (2) Univariable LMMs^aj^ to predict EPDS scores using all features as fixed effects, participant ID as a random intercept, and family income and maternal age as covariates. Benjamini–Hochberg False Discovery Rate correction to adjust *P* values, with *q*<0.05 and *q*<0.1 considered significant.	Standardized regression coefficient (β), 95% CIs, *P* values, and corrected *q* values	PPD associated with higher baby crying counts on weekdays (β=+.2092; *q*<0.05), longer stay duration at a single location during the night (β=+.2962; *q*<0.05), and more total active bouts in weekday afternoons (β=−0.2436; *q*<0.05). Greater median travel distance on weekday mornings was potentially associated with increased depressive symptoms (0.05≤*q*<0.10).

a PHQ-9: Patient Health Questionnaire-9.

b GAD-7: Generalized Anxiety Disorder-7.

c HR: heart rate.

d HRV: heart rate variability.

e RF: random forest.

f GLM: generalized linear model.

g SVM: support vector machine.

h KNN: k-nearest neighbors.

i SHAP: Shapley Additive Explanations.

j mAUC: multiclass area under the curve.

k BMR: basal metabolic rate.

l PPD: postpartum depression.

m CAML: Context-Aware Adaptive Meta-Learning.

n LSTM: long short-term memory.

o CNN: convolutional neural network.

p XGBoost: Extreme Gradient Boosting.

q LIME: Local Interpretable Model-Agnostic Explanations.

r IBI: interbeat interval.

s AUC: area under the curve.

t LR: logistic regression.

u NB: Naïve Bayes.

v SGD: stochastic gradient descent.

w DT: decision tree.

x GB: gradient boosting.

y ANN: artificial neural network.

z CVRR: coefficient of variation of RR intervals.

aa RMSSD: root-mean-square of successive differences between RR intervals.

ab SDNN: SD of NN intervals.

ac HF: power of the high-frequency band.

ad LF: power of the low-frequency band.

ae pNN50: percentage of successive RR intervals that differ by more than 50 ms.

af NN50: successive RR intervals that differ by more than 50 ms.

ag EMA: ecological momentary assessment.

ah AVNN: average of NN intervals.

ai EPDS: Edinburgh Postnatal Depression Scale.

aj LMM: linear mixed effects model.

#### Perinatal Depression

Faherty et al [33] used generalized linear mixed effects models to examine associations between movement patterns and both self-reported mood and depression or anxiety scores. They found that the radius of travel was significantly associated with depression severity. Women with more severe depression had a median daily radius of 1.9 miles versus 2.7 miles in milder cases (*P*=.04). A worsening of daily mood from the prior day was associated with a 5% contraction in radius (*P*=.03), and each PHQ-9 point corresponded to a 64% smaller radius around assessment days (*P*=.003).

Hurwitz et al [34] used individualized multinomial machine learning classification models to classify 4 perinatal periods: prepregnancy, pregnancy, postpartum without depression, and PPD. The individualized random forest models using Fitbit-derived features distinguished these 4 periods with a mean multiclass area under the curve (AUC; mAUC) of 0.85 and Cohen κ of 0.80, outperforming generalized linear models (mAUC=0.82), support vector machines (mAUC=0.75), and k-nearest neighbors (mAUC=0.74). For the PPD class specifically, the model achieved a sensitivity of 0.79, a specificity of 0.95, a precision of 0.84, and an *F*_1_-score of 0.81.

Li et al [24] reported a weighted risk scoring framework combining self-report scales with app-derived phenotypic scores estimated using multivariable logistic regression to classify PPD risk as low, moderate, or high. At 3 weeks postpartum, the multimodal model achieved 90.0% sensitivity, 84.1% specificity, and an AUC of 0.871. The study also showed that the mobile health intervention, a smartphone app integrating continuous self-report with passively collected behavioral data to generate automated PPD risk alerts, shortened the mean time to PPD identification from 26.9 days under standard care (ie, scheduled screenings) to 11.8 days in the intervention group (*P*<.001).

Tang et al [22] used feature selection followed by univariable linear mixed models to predict EPDS scores and identify behavioral features associated with PPD symptoms. They identified 8 behavioral features significantly associated with weekly EPDS scores after false discovery rate correction. Baby crying count on weekdays (β*=+*.21; *q*<0.05) and maximum nighttime GPS stay duration (β=+.30; *q*<0.05) were positively associated with depressive symptom burden, while afternoon active bouts on weekdays (β=−0.24; *q*<0.05) and afternoon stationary bout count (β=−0.23; *q*<0.05) were negatively associated. The study further identified a potentially stress-inducing greater travel distance on weekday morning pattern (β=+.17; *q*<0.10), contrasting with the typically inverse relationship between mobility and depression observed in nonpostpartum populations [50,51].

Jose et al [23] used a meta-learning framework combining long short-term memory networks, convolutional neural networks, and gradient boosting models to classify PPD risk levels, reporting a predictive accuracy of 98.7%, the highest figure reported in the included literature. However, this result should be interpreted cautiously, as key methodological details, including the wearable devices used, the mental health outcome measures, and the validation procedures, were insufficiently reported in this conference paper.

Singh Solorzano et al [38] used Pearson correlation and hierarchical linear regression to examine whether prepartum RMSSD was associated with and predicted postpartum depressive symptoms. They found that lower prepartum RMSSD independently predicted depressive symptoms 1 month postpartum (β=−0.22; *P*=.01), after adjustment for prepartum depressive symptoms and potential confounders. These findings suggest that pregnancy may represent a potentially important window for early risk stratification and preventive monitoring.

#### Prenatal Stress

King et al [36] developed the microstress EMA framework to identify a micro-EMA item aligned with physiological stress for labeling passively sensed data in pregnant women. Using laboratory data from 18 nonpregnant women, they trained stress classifiers on wearable ECG or interbeat interval–derived features before applying the model to in-the-wild recordings from pregnant women. “WorriedStress” showed the most promise as a short-window micro-EMA label. However, as the model was developed in a nonpregnant laboratory sample and evaluated in a separate pregnant field sample, the findings are best interpreted as proof-of-concept transfer across populations and contexts rather than as externally validated perinatal stress prediction.

Ng et al [35] used machine learning classification models to predict next-day physiological and perceived stress in a cohort of 16 pregnant individuals. Random forest classifiers achieved an *F*_1_-score of 0.84 for physiological stress and 0.74 for perceived stress. A notable observation was that physiological and perceived stress appeared to rely on different predictive signals: when EMA features were excluded, performance remained comparable for physiological stress but was substantially reduced for perceived stress.

#### Emotional States in Pregnancy

Li et al [39] used machine learning classification models to predict 4 discrete emotional states (happy, anxious, sad, and frustrated) among 53 pregnant women across gestational weeks 23 to 32. Among the 10 machine learning classifiers evaluated, random forest achieved the highest area under the receiver operating characteristic curve of 0.70 for multiclass emotion prediction.

#### Maternal Loneliness

Sarhaddi et al [37] used machine learning classification models to classify maternal social loneliness status during pregnancy and the postpartum period using objective sensor data. Gradient boosting achieved a weighted *F*_1_-score of 0.897, and decision tree achieved a weighted *F*_1_-score of 0.872 based on smartwatch-derived HRV, sleep, and physical activity data from 39 data samples contributed by 31 participants. Adding sleep features to physical activity features slightly reduced classification performance among participants classified as lonely.

#### Validation Strategies

Reported model performance should be interpreted in the context of the validation strategies used, as high AUC, *F*_1_-score, or accuracy values in small datasets may reflect overfitting, nonindependent observations, or optimistic validation rather than generalizable performance.

Internal resampling was the main validation approach among studies that developed predictive classifiers. Hurwitz et al [34] used 3 repeats of 10-fold cross-validation within individualized models; each model was developed and tested using data from a single participant. Ng et al [35] used 5-fold cross-validation with folds randomly drawn from all participant-days combined, and Li et al [39] similarly randomly split pooled observations into training and testing sets under 5-fold cross-validation; in both studies, repeated observations from the same individual could therefore fall into both partitions. In contrast, Sarhaddi et al [37] used leave-one-participant-out cross-validation, keeping observations from the same individual entirely within either the training or the testing set. Jose et al [23] used k-fold cross-validation but did not report whether folds were separated at the participant level.

One study (King et al [36]) used separate-sample validation, but the design involved 2 simultaneous shifts rather than conventional external validation. They trained the model on laboratory-induced stress data from nonpregnant women and then applied it to in-the-wild recordings from a separate group of pregnant women. This should therefore be interpreted as a proof-of-concept transfer across both populations and contexts, rather than as external validation in an independent perinatal cohort or as participant-level internal validation.

No study performed external validation in an independent cohort. The only related analysis was conducted by Hurwitz et al [34], who trained individualized models in participants with PPD and used a non-PPD control group from the same dataset as a specificity check. Finally, no study assessed calibration; model performance was reported using discrimination and classification metrics only. Taken together, the high discrimination metrics reported above should be interpreted cautiously.

### Explainability and Features Reported as Useful

Across the reviewed studies, several approaches were used to interpret model outputs and identify the physiological and behavioral features most relevant to perinatal mental health outcomes.

#### Use of Explainability Techniques

Three studies integrated post hoc explainable AI (XAI) routines alongside their predictive pipelines. XAI refers to AI systems that, given a target audience, provide details or reasons that make their functioning clearer or easier to understand [52]. In applied predictive modeling, commonly used XAI techniques often aim to improve the interpretability of complex or “black box” machine learning models by estimating how input features contribute to model outputs. Jose et al [23] embedded both Shapley Additive Explanations (SHAP) [53] and Local Interpretable Model-Agnostic Explanations [54] within their Context-Aware Adaptive Meta-Learning framework for PPD risk estimation. Ng et al [35] applied SHAP to their random forest models to provide both global feature importance rankings and local, instance-level explanations for stress prediction. Hurwitz et al [34] combined SHAP with a permutation-based variable importance method to rank candidate features associated with PPD across individualized random forest models, using SHAP dependence plots to visualize the direction and magnitude of each feature’s relationship with the PPD class.

Other studies used more limited model-intrinsic or statistical interpretability approaches. Sarhaddi et al [37] examined decision tree structures and Gini-based feature importance from gradient boosting models to identify smartwatch-derived predictors of maternal social loneliness. Li et al [39] ranked 9 HRV indicators using random forest feature importance for predicting 4 emotional states, reporting that performance plateaued after the top 5 features were included. Tang et al [22] applied regularization-based feature selection followed by linear mixed effects models to identify passive sensing features associated with EPDS scores. In 2 further studies, interpretability was based on classical statistical modeling: Faherty et al [33] applied generalized linear mixed effects regression to examine associations between mood and mobility features, and Li et al [24] used multivariable logistic regression to identify independent features of PPD within a mobile health digital phenotyping model.

#### Features Reported as Useful

Across the included studies, a range of features were reported as useful for detecting or predicting perinatal mental health outcomes. These features mainly fell into 2 categories: physiological features, derived predominantly from cardiac signals, such as HRV features; and behavioral and contextual features, such as physical activity, sleep, and device usage patterns. As the included studies differed substantially in devices, feature-engineering pipelines, outcomes, time windows, and modeling approaches, feature importance was not directly comparable across studies. We therefore summarized these 2 categories of features reported as useful within each study, together with the criteria used, in Table 4.

**Table 4.** Physiological features and behavioral and contextual features reported as useful and the criterion used.

Study	Physiological features	Behavioral and contextual features	Criteria used
Faherty et al, 2017 [33]	—^a^	Travel radius	Generalized linear mixed-effects regression coefficients
Hurwitz et al, 2024 [34]	Average HR^b^, first-quartile HR, and minimum HR	Calories BMR^c^, lightly active minutes	SHAP^d^ and permutation feature importance
Li et al, 2025 [24]	—	Sleep duration, sleep efficiency, physical activity, social activity radius, and speaking rate	Multivariable logistic regression coefficients
Li et al, 2022 [39]	CVRR^e^, RMSSD^f^, SDNN^g^, HF^h^, LF^i^/(LF+HF)	—	Random forest feature importance
Ng et al, 2022 [35]	HRV^j^-derived duration-based features: consecutive stress minutes and stress-minute percentage	Number of children	SHAP feature importance
Sarhaddi et al, 2023 [37]	Resting SDNN, LF, LF/HF, AVNN^k^, and resting HR	Activity intensity, steps kurtosis during the day, activity duration, step and activity distribution, and sedentary time	Decision tree and gradient boosting feature importance
Singh Solorzano et al, 2022 [38]	Prepartum RMSSD	—	Pearson correlation and hierarchical linear regression coefficient
Tang et al, 2025 [22]	—	Weekday baby crying count, GPS stay duration at a single location during the night, total active bouts in weekday afternoons, and travel distance on weekday mornings	Linear mixed model coefficient

a Not applicable.

b HR: heart rate.

c BMR: basal metabolic rate.

d SHAP: Shapley Additive Explanations.

e CVRR: coefficient of variation of RR intervals.

f RMSSD: root-mean-square of successive differences between RR intervals.

g SDNN: SD of NN intervals.

h HF: power of the high-frequency band.

i LF: power of the low-frequency band.

j HRV: heart rate variability.

k AVNN: average of NN intervals.

Across studies examining physiological features, HRV-related features were examined most frequently, reflecting ANS function and its association with depression risk and severity [18,55]. Li et al [39] reported that the CVRR, RMSSD, SDNN, HF, and LF power were among the most important HRV features in their random forest models for detecting maternal emotions. Singh Solorzano et al [38] found that lower prepartum RMSSD independently predicted higher postpartum depressive symptoms after adjustment for baseline depression and covariates. In models of maternal social loneliness, Sarhaddi et al [37] highlighted resting HR, resting SDNN, LF, ratio of LF-to-HF power (LF/HF), and average NN intervals as key contributors alongside activity-related features. In individualized PPD prediction models, Hurwitz et al [34] identified average HR, first-quartile HR, and minimum HR as key contributors.

Behavioral and contextual features derived from smartphones and wearables were also frequently reported as useful, particularly those capturing activity, mobility, sleep, and daily routines. Sarhaddi et al [37] identified physical activity intensity and the kurtosis of hourly step counts as the most important features in their models of maternal social loneliness, with additional contributions from distributional statistics of step count, activity duration, and sedentary time. Faherty et al [33] found that the radius of travel was most sensitive to mood, with worsening mood and higher PHQ-9 scores associated with reduced mobility range. Tang et al [22] reported that infant-related sleep disruptions, increased morning crying events, longer nighttime stay duration, reduced afternoon activity, and altered mobility patterns were associated with higher EPDS scores.

### Quality Assessment

The methodological quality and risk of bias were assessed using PROBAST+AI, with model development and evaluation assessed separately. Faherty et al [33] and Tang et al [22] were considered not applicable for assessment using PROBAST+AI because they examined associations between sensing features and mental health outcomes without developing or evaluating an individual-level prediction model. Among the remaining studies, most contributed 2 assessment units, one for development and one for evaluation, whereas King et al [36] contributed only an evaluation unit because its model was developed in a nonpregnant sample and evaluated in a perinatal population. This yielded 15 assessment units (Table 5).

**Table 5.** Quality assessment results.

Study	Type	Quality concern or risk of bias				Applicability			Overall	
		Participants	Predictors	Outcome	Analysis	Participants	Predictors	Outcome	Risk of bias	Applicability
Hurwitz et al, 2024 [34]	Dev^a^	?^b^	-^c^	+^d^	-	?	-	+	-	-
Hurwitz et al, 2024 [34]	Eva^e^	?	-	+	-	?	-	+	-	-
Jose et al, 2025 [23]	Dev	?	+	+	-	?	+	+	-	?
Jose et al, 2025 [23]	Eva	?	+	+	-	?	+	+	-	?
King et al, 2019 [36]	Eva	?	?	-	-	?	?	-	-	-
Li et al, 2025 [24]	Dev	+	+	+	+	+	+	+	+	+
Li et al, 2025 [24]	Eva	+	+	+	-	+	+	+	-	+
Li et al, 2022 [39]	Dev	+	+	-	-	+	+	+	-	+
Li et al, 2022 [39]	Eva	+	+	-	-	+	+	+	-	+
Ng et al, 2022 [35]	Dev	+	+	+	-	+	+	+	-	+
Ng et al, 2022 [35]	Eva	+	+	+	-	+	+	+	-	+
Sarhaddi et al, 2023 [37]	Dev	+	+	+	-	+	+	+	-	+
Sarhaddi et al, 2023 [37]	Eva	+	+	+	-	+	+	+	-	+
Singh Solorzano et al, 2022 [38]	Dev	+	+	+	+	+	+	+	+	+
Singh Solorzano et al, 2022 [38]	Eva	+	+	+	+	+	+	+	+	+

a Dev: development.

b ?: unclear.

c -: high concern for quality or high risk of bias

d +: low concern for quality or low risk of bias.

e Eva: evaluation.

Overall, 3 of the 15 assessment units were rated as low overall quality concern or risk of bias, whereas 12 were rated as having a high overall quality concern or risk of bias. Across domains, the participants domain showed unclear judgments in 5 units due to insufficient reporting, whereas the predictors and outcome domains were relatively robust, with few high-risk ratings. The analysis domain was the primary source of concern, with 12 of 15 units rated as high concern or high risk of bias. Common issues included small sample sizes relative to model complexity, limited or absent validation, lack of calibration assessment, inadequate handling of missing data, and optimistic performance reporting without correction for overfitting. These concerns informed the interpretation of model performance. Reported performance metrics were therefore interpreted as preliminary, largely internally derived estimates, rather than as evidence of established predictive performance or clinical utility.

## Discussion

### Principal Findings

#### Background

Perinatal mental health conditions affect approximately 20% of pregnant and postpartum individuals and are associated with a broad spectrum of adverse outcomes, including significant functional impairment, increased risk of chronic psychiatric disorders, and increased suicidality. These conditions can also disrupt mother-infant bonding and are linked to adverse developmental outcomes in offspring. The scale and severity of these impacts, together with persistent barriers to timely care during the perinatal period, underscore the potential value of continuous, real-world monitoring using mobile sensing technologies.

This systematic review examined the use of smartphones and wearable devices to detect and predict perinatal mental health outcomes. Overall, the findings suggest that mobile sensing shows preliminary potential for perinatal mental health assessment, but the current evidence base remains limited by narrow outcome coverage, restricted technological approaches, and substantial methodological weaknesses.

This section first synthesizes 3 core observations emerging from the included studies and then situates the current evidence base within the broader perinatal mental health and digital phenotyping literature to characterize the scope of mental health outcomes examined and the technological approaches represented to date.

#### Summary of Key Observations

The included studies converge on 3 key observations. First, ANS-related physiological features, particularly HR and HRV features, were frequently reported as important model contributors within individual studies. Li et al [39] reported that CVRR, RMSSD, SDNN, HF, and LF contributed to the detection of maternal emotions. Sarhaddi et al [37] highlighted resting HR, resting SDNN, LF, LF/HF, and average NN intervals as key contributors to maternal loneliness prediction. Hurwitz et al [34] identified average HR, first-quartile HR, and minimum HR as key contributors in individualized PPD prediction models. The relevance between these autonomic features and mental health is consistent with the neurovisceral integration model, which links vagally mediated HRV to the functional integrity of prefrontal-subcortical circuits involved in emotion regulation [56].

Notably, Singh Solorzano et al [38] found that lower prepartum RMSSD independently predicted higher depressive symptoms at 1 month postpartum after adjustment for baseline depression and covariates. This finding is supported by evidence from nonperinatal populations showing that reduced vagal tone is associated with depression, anxiety, and impaired stress reactivity [18,55]. This finding further suggests that ANS activity during pregnancy may provide information about subsequent postpartum mood outcomes, rather than merely reflecting concurrent emotional states. One possible explanation is that pregnancy is characterized by substantial autonomic adaptation to support maternal-fetal hemodynamic demands [57] and that disrupted autonomic adaptation or reduced parasympathetic reserve may be expressed both as altered HRV during pregnancy and as greater vulnerability to postpartum mood symptoms.

Second, behavioral and contextual features were also reported as useful for detecting and predicting perinatal mental health outcomes, and their interpretation appears to require context-specific consideration within perinatal populations rather than direct extrapolation from general population findings. Sarhaddi et al [37] identified physical activity intensity and hourly step-count kurtosis as key features for maternal social loneliness. Faherty et al [33] found reduced radius of travel to be associated with worsening mood and higher PHQ-9 scores. Tang et al [22] reported that infant-related sleep disruptions, increased morning crying events, longer nighttime stay duration, reduced afternoon activity, and altered mobility patterns were associated with higher EPDS scores. These findings further indicate that behavioral features may provide meaningful information about mental health [7].

In nonperinatal adults, reduced mobility, increased home dwell time, and disrupted circadian patterns are well-established correlates of depressive symptoms [50,51]. Faherty et al [33] replicated this pattern, with a reduced radius of travel tracking worsening mood in pregnant women. However, Tang et al [22] identified an inverse association during the postpartum period: greater weekday-morning travel distance was associated with higher EPDS scores. This divergence likely reflects the distinct behavioral context of early postpartum life, in which increased mobility may indicate infant care demands, medical visits, or disrupted routines rather than behavioral activation. Similarly, longer nighttime GPS stay duration, typically indicative of healthy rest in general populations, was associated with higher symptom burden [22], possibly reflecting prolonged sedentary awakenings related to infant care. These findings further emphasize the need to interpret passive sensing features within context, rather than assuming that feature-outcome relationships generalize across life stages [58].

Third, the limited available evidence suggests that multimodal and individualized modeling strategies may offer advantages over population-level, single-modality approaches. On the multimodal side, the 2 highest-performing PPD models in this review both integrated heterogeneous inputs: Jose et al [23] integrated passive sensing data (HR, HRV, sleep quality, and physical activity) with other app-derived data (social interaction logs; collection method not reported) and clinical data (prenatal, delivery, and postnatal examination records) within a meta-learning framework, and Li et al [24] combined passive sensing data (phone usage features), other app-derived data (voice features extracted from voice diaries), and active self-report data (weekly self-reported scales and daily diaries on mood, sleep, breastfeeding, and infant interaction). These patterns indicate that stronger reported performance may be related to the incorporation of data sources beyond passive sensing alone while also highlighting potential trade-offs between predictive performance and implementation demands across data source categories. Different data source categories have distinct implementation implications. Passive sensing can reduce deliberate input, but background collection can still involve technical burden, device-dependent or operating system–dependent missingness, and privacy concerns related to behavioral traces [59]. Active self-report inputs may improve predictive performance but reintroduce participant burden, particularly in the perinatal period, when sleep disruption and caregiving demands may limit engagement with self-monitoring [60]. Other app-derived data, such as social interaction logs and voice-derived features, may capture social or affective functioning but depend on app engagement and raise privacy concerns if they reveal identity, relationships, or sensitive affective information [61]. Clinical data may add contextual information, but their availability and completeness depend on documentation practices, access to care, and record linkage, which may introduce selection bias and limit scalability [62].

On the personalization side, Hurwitz et al [34] reported strong discrimination of PPD using individualized random forest models under internal validation, consistent with findings in nonperinatal populations that within-person deviations may be more informative than between-person comparisons [63]. Although only one included study implemented an individualized approach, the perinatal period, with its rapid within-person physiological and behavioral changes, is a context in which personalized modeling is likely to offer substantial advantages. Its limited use, therefore, represents a clear methodological gap.

Beyond these methodological observations, placing the current evidence base within the broader perinatal mental health and digital phenotyping literature highlights additional gaps in what has been studied. Two are particularly notable: the limited range of mental health outcomes examined and the restricted use of available sensing and analytical technologies.

#### Perinatal Mental Health Outcomes: Coverage and Gaps

The 10 included studies converged on a narrow set of outcomes. PPD dominated the evidence base (6 studies), followed by stress (2 studies), discrete emotions (1 study), and maternal social loneliness (1 study); anxiety appeared only as a co-tracked outcome rather than a primary target. This pattern reflects the longstanding clinical and research emphasis on PPD, while leaving substantial areas of perinatal psychopathology largely unexamined within the digital sensing literature.

Several clinically important conditions are notably underrepresented. Perinatal anxiety disorders affect 15% to 20% of pregnant and postpartum individuals [64,65], a prevalence comparable to depression, but were not examined as a primary outcome in any of the included studies. This represents a missed opportunity rather than a methodological barrier: anxiety is associated with physiological and behavioral features, such as elevated sympathetic tone, fragmented sleep, and nighttime phone use, that can be captured by current passive sensing pipelines and have already been studied in nonperinatal populations [12]. Fear of childbirth is another increasingly recognized perinatal concern, associated with obstetric interventions, such as cesarean section, poorer birth experience, and elevated risks of postpartum depression, anxiety, and posttraumatic stress disorder [66], yet none of the reviewed studies targeted this outcome.

Beyond these, the evidence was also silent on trauma-related disorders, particularly childbirth-related posttraumatic stress disorder [67], as well as perinatal substance and alcohol use disorders [68,69], perinatal obsessive-compulsive disorder [70], bipolar disorder [71], and eating disorders [72]. Moreover, almost all studies modeled outcomes in isolation, rather than addressing the high comorbidity that characterizes perinatal psychopathology.

#### Technological Approaches: Coverage and Gaps

The technological choices made across the included studies reflect only a narrow slice of the methods now increasingly used in digital mental health and wearable biosignal processing. In terms of signal modality, the majority of studies relied on traditional physiological and behavioral features, such as HRV, mobility, sleep, and screen use, with Li et al [24] being the only study to incorporate voice-derived features. In terms of modeling approach, analyses were dominated by tree-based ensembles and classical regression. In terms of training paradigm, most models were trained from scratch on relatively small, study-specific datasets, without using pretrained representations from external biosignal corpora.

These choices leave several gaps relative to the methodological possibilities now available in the broader field. In terms of modality coverage, voice and language have become well-established streams in digital mental health phenotyping. Acoustic features, such as speaking rate, pitch variability, and pause frequency, as well as linguistic content from free text, are particularly informative for anxiety and trauma-related symptoms [73,74]. In terms of temporal modeling, the perinatal period is characterized by pronounced and sustained change, including autonomic adaptation, progressive sleep disruption, and evolving caregiving demands. However, models based on aggregated weekly or daily features and applied within cross-sectional or short-window classification frameworks can capture this temporal structure only to a limited extent [63,75]; the most clinically relevant signal may lie in the trajectory of change rather than in any single observation window. In terms of data scale and training paradigm, perinatal cohorts are inherently difficult to recruit and retain in sufficient numbers for end-to-end supervised learning [76], and the resulting small sample sizes raise well-documented risks of overfitting and unstable performance estimates [77,78]. This represents a structural mismatch rather than merely a resource limitation, particularly as the field moves beyond feasibility demonstration toward generalizable clinical utility.

### Limitations

While the findings mentioned earlier highlight the emerging potential of mobile sensing for perinatal mental health, several limitations at both the review and individual study levels warrant careful consideration.

#### Limitations of the Included Studies

The field remains at an early stage of development: only 10 studies met the inclusion criteria, and several methodological constraints limit the strength of the current evidence. First, sample sizes were consistently small relative to model complexity, with 5 of 10 studies enrolling fewer than 40 participants [22,33,35-37], raising risks of overfitting and optimistic performance estimates [77,78].

Second, validation designs were generally limited and susceptible to optimistic performance estimates. External validation was largely absent; no study validated a model in an independent perinatal cohort. Given that health AI models may perform worse when applied to populations that differ in cultural, caregiving, and health care contexts, this limits confidence that the reported models would generalize across heterogeneous perinatal populations, care settings, or stages of pregnancy and postpartum.

Third, observation windows were often short, with several studies collecting data for 4 weeks or less. As perinatal mental health follows trajectory-based onset rather than static states [79], such short windows limit inference about precisely the temporal dynamics digital sensing is best placed to capture.

Fourth, methodological reporting was often insufficient to evaluate model quality: handling of missing data, hyperparameter tuning, and validation strategies were frequently underspecified, and few studies followed standardized frameworks such as TRIPOD+AI (Transparent Reporting of a Multivariable Prediction Model for Individual Prognosis or Diagnosis With AI) [80]. This was reflected in the PROBAST+AI assessment, where 80% (12/15) of assessment units were rated at high overall quality concern or risk of bias, driven primarily by the analysis domain, which further limits confidence in the reported performance.

Fifth, although most studies used validated scales to measure self-report outcomes, 2 studies used nonvalidated outcome measures; their performance estimates should therefore be interpreted cautiously and not as evidence of prediction against established clinical or psychometric criteria.

#### Limitations of the Review

Several limitations of the review process should also be acknowledged. First, substantial heterogeneity across devices, derived features, analytical pipelines, and outcome definitions precluded meta-analysis, necessitating a narrative synthesis. As a result, we could summarize patterns across studies but could not provide pooled estimates of model performance or formally quantify between-study differences.

Second, our search strategy may have introduced bias: searches in databases were restricted to title and abstract fields, which may have missed studies in which perinatal status, mental health outcomes, or sensing methods were described only in keywords or full text. We also did not include gray literature, preprints, or unpublished trials, and restricting the search to English-language publications may have excluded relevant studies and introduced language bias. Given that only 10 studies were included, even a small number of missed studies could have affected the synthesis and the strength of the conclusions.

Third, data extraction was checked by a second reviewer for a randomly selected 40% (4/10) of the included studies rather than for all studies. Although agreement was high and discrepancies were minor and nonsubstantive, full independent verification was not conducted for every study; therefore, a small risk of undetected extraction errors cannot be entirely excluded.

### Future Directions

The limitations identified earlier translate into several priorities for subsequent research. Regarding mental health outcome coverage, future research should expand beyond PPD to address the broader spectrum of perinatal mental health conditions. In particular, perinatal anxiety disorders, trauma-related disorders, perinatal obsessive-compulsive disorder, bipolar disorder, and eating disorders remain substantially understudied despite their clinical significance. Given that many of these conditions may manifest through measurable physiological and behavioral changes, they represent feasible targets for passive sensing approaches. Future studies should also move beyond modeling isolated outcomes and consider transdiagnostic or multimorbidity-aware frameworks that better reflect the high comorbidity characteristic of perinatal psychopathology.

Regarding technological approaches, several methodological opportunities require further investigation. For signal modalities, voice and language data may provide additional information when collected in appropriate clinical or app-based contexts, such as consultation recordings or free-text entries. These data could be analyzed using pretrained speech encoders or language models to extract acoustic and linguistic markers of depression and anxiety [73,74]. For temporal modeling, sequence-aware architectures, such as temporal convolutional networks and time series transformers [81], may help capture gradual autonomic and behavioral changes when sufficient longitudinal data are available. Within-person normalization, as demonstrated by Hurwitz et al [34], represents a complementary strategy, addressing the same challenge by anchoring inference to individual longitudinal baselines rather than cross-sectional cohort averages. For data scarcity, foundation models pretrained on large unlabeled wearable datasets [82,83] may reduce the need to train models entirely from scratch, although their value in perinatal populations remains to be established.

Regarding methodological and study design improvements, recruitment strategies and sample sizes should be calibrated to the complexity of the modeling task, guided by established sample size frameworks for clinical prediction models [84] rather than by feasibility alone. Preregistered analysis plans, which are rare in the current literature, would constrain post hoc feature selection and reduce optimistic bias, while standardized reporting via TRIPOD+AI [80] would enable cross-study comparison and cumulative synthesis. Longitudinal designs spanning prepregnancy through 1 year postpartum are also needed to characterize the autonomic and behavioral trajectories that precede symptom onset and to identify the optimal timing for preventive intervention. Combining passive sensing with biological measures, such as inflammatory markers or hypothalamic-pituitary-adrenal axis features, may help clarify mechanisms linking perinatal physiology, behavior, and mood [85]. Finally, human-centered design studies should examine how pregnant and postpartum individuals themselves perceive continuous monitoring, including concerns around privacy, data ownership, and the psychological impact of being algorithmically assessed during a vulnerable life phase [86,87].

### Conclusions

This systematic review synthesizes evidence on the use of wearable devices and smartphones for detecting and predicting perinatal mental health outcomes. Overall, the evidence suggests preliminary promise but remains insufficient to support clinical implementation. HRV-derived physiological features and behavioral and contextual features related to mobility, physical activity, and sleep were frequently examined or reported as useful within individual studies, and their interpretation requires perinatal-specific contextualization. Despite this preliminary promise, the field faces 3 critical constraints. Methodologically, 80% (12/15) of assessment units were rated as having a high overall quality concern or risk of bias, and independent external validation in perinatal populations is currently lacking. In terms of outcome scope, research has focused primarily on PPD, while anxiety and other prevalent perinatal mental health conditions remain largely underexamined. Technologically, the sensing modalities and analytical approaches used to date represent only a narrow subset of currently available methods. Given these limitations, mobile sensing tools should not yet be used for clinical screening or decision-making in perinatal mental health. Realizing clinical potential will require expanded outcome and modality coverage, larger longitudinal cohorts with preregistered analysis plans, standardized reporting aligned with frameworks such as TRIPOD+AI, incorporation of sequence-aware and pretrained modeling approaches suited to perinatal trajectories, stronger validation strategies, and human-centered designs that address the privacy, burden, and psychological implications of continuous monitoring during this uniquely vulnerable life phase.

## Supplementary material

10.2196/101677 Multimedia Appendix 1 Full search strategies for all databases.

10.2196/101677 Checklist 1 PRISMA 2020 checklist.

## Abbreviations

ANS: autonomic nervous system
AUC: area under the curve
CVRR: coefficient of variation of RR interval
ECG: electrocardiogram
EMA: ecological momentary assessment
EPDS: Edinburgh Postnatal Depression Scale
HF: high frequency
HR: heart rate
HRV: heart rate variability
LF: low frequency
mAUC: multiclass area under the curve
PHQ: Patient Health Questionnaire
PICO: population, intervention, comparator, and outcome
PPD: postpartum depression
PPG: photoplethysmography
PRISMA: Preferred Reporting Items for Systematic Reviews and Meta-Analyses
PROBAST+AI: Prediction Model Risk of Bias Assessment Tool With AI extension
PROSPERO: International Prospective Register of Systematic Reviews
RMSSD: root-mean-square of successive difference
SDNN: standard deviation of NN interval
SHAP: Shapley Additive Explanations
SPIDER: sample, phenomenon of interest, design, evaluation, and research type
TRIPOD+AI: Transparent Reporting of a Multivariable Prediction Model for Individual Prognosis or Diagnosis With AI
WHO: World Health Organization
XAI: explainable AI
