# Supplementary material for: Perinatal Mental Health Detection and Prediction Using Mobile Sensing Data: Systematic Review
Source: JMIR Ment Health. 2026 Sep 25;13:e101677. doi: 10.2196/101677 (PMC13614507; doi:10.2196/101677)
Supplement: Supplementary file 1 [file mental-v13-e101677-s001.pdf]

# Full Search Strategies for All Databases

## Initial search

• Database: PubMed
• Search string: (pregnan*[tiab] OR perinatal[tiab] OR antenatal[tiab] OR prenatal[tiab] OR postnatal[tiab] OR postpartum[tiab] OR maternal*[tiab]) AND (depression*[tiab] OR anxiet*[tiab] OR stress*[tiab] OR trauma*[tiab] OR suicid*[tiab] OR self-harm*[tiab] OR phobia*[tiab] OR emotion*[tiab] OR distress[tiab] OR mental*[tiab] OR psychiatric[tiab]) AND (wearable*[tiab] OR sensor*[tiab] OR smartphone*[tiab] OR "mobile phone*"[tiab] OR "mobile app*"[tiab] OR mhealth[tiab] OR smartwatch*[tiab] OR "digital phenotyping"[tiab] OR "passive sensing"[tiab]) AND (predict*[tiab] OR forecast*[tiab] OR detect*[tiab] OR monitor*[tiab] OR track*[tiab] OR model*[tiab] OR algorithm*[tiab] OR "machine learning"[tiab])
• Date when made searches: 2026/01/14
• Limits: none

• Database: Web of Science
• Search string: (TI=(pregnan* OR perinatal OR antenatal OR prenatal OR postnatal OR postpartum OR maternal*) OR AB=(pregnan* OR perinatal OR antenatal OR prenatal OR postnatal OR postpartum OR maternal*)) AND (TI=(depression* OR anxiet* OR stress* OR trauma* OR suicid* OR self-harm* OR phobia* OR emotion* OR distress OR mental* OR psychiatric) OR AB=(depression* OR anxiet* OR stress* OR trauma* OR suicid* OR self-harm* OR phobia* OR emotion* OR distress OR mental* OR psychiatric)) AND (TI=(wearable* OR sensor* OR smartphone* OR "mobile phone*" OR "mobile app*" OR mhealth OR smartwatch* OR "digital phenotyping" OR "passive sensing") OR AB=(wearable* OR sensor* OR smartphone* OR "mobile phone*" OR "mobile app*" OR mhealth OR smartwatch* OR "digital phenotyping" OR "passive sensing")) AND (TI=(predict* OR forecast* OR detect* OR monitor* OR track* OR model* OR algorithm* OR "machine learning") OR AB=(predict* OR forecast* OR detect* OR monitor* OR track* OR model* OR algorithm* OR "machine learning"))
• Date when made searches: 2026/01/14
• Limits: none

• Database: Scopus
• Search string: TITLE-ABS ( ( pregnan* OR perinatal OR antenatal OR prenatal OR postnatal OR postpartum OR maternal* ) AND ( depression* OR anxiet* OR stress* OR trauma* OR suicid* OR self-harm* OR phobia* OR emotion* OR distress OR mental* OR psychiatric ) AND ( wearable* OR sensor* OR smartphone* OR "mobile phone*" OR "mobile app*" OR mhealth OR smartwatch* OR "digital phenotyping" OR "passive sensing" ) AND ( predict* OR forecast* OR detect* OR monitor* OR track* OR model* OR algorithm* OR "machine learning" ) )
• Date when made searches: 2026/01/14
• Limits: none

• Database: APA PsycInfo
• Search string: (TI(pregnan* OR perinatal OR antenatal OR prenatal OR postnatal OR postpartum OR maternal*)  OR AB(pregnan* OR perinatal OR antenatal OR prenatal OR postnatal OR postpartum OR

maternal*)) AND (TI(depression* OR anxiet* OR stress* OR trauma* OR suicid* OR self-harm* OR phobia* OR emotion* OR distress OR mental* OR psychiatric) OR AB(depression* OR anxiet* OR stress* OR trauma* OR suicid* OR self-harm* OR phobia* OR emotion* OR distress OR mental* OR psychiatric)) AND (TI(wearable* OR sensor* OR smartphone* OR "mobile phone*" OR "mobile app*" OR mhealth OR smartwatch* OR "digital phenotyping" OR "passive sensing") OR AB(wearable* OR sensor* OR smartphone* OR "mobile phone*" OR "mobile app*" OR mhealth OR smartwatch* OR "digital phenotyping" OR "passive sensing")) AND (TI(predict* OR forecast* OR detect* OR monitor* OR track* OR model* OR algorithm* OR "machine learning") OR AB(predict* OR forecast* OR detect* OR monitor* OR track* OR model* OR algorithm* OR "machine learning"))
• Date when made searches: 2026/01/14
• Limits: none

• Database: IEEE Xplore
• Search string: (pregnan* OR perinatal OR antenatal OR prenatal OR postnatal OR postpartum OR maternal*) AND (depression* OR anxiet* OR stress* OR trauma* OR suicid* OR self-harm* OR phobia* OR emotion* OR distress OR mental* OR psychiatric) AND (wearable* OR sensor* OR smartphone* OR "mobile phone*" OR "mobile app*" OR mhealth OR smartwatch* OR "digital phenotyping" OR "passive sensing") AND (predict* OR forecast* OR detect* OR monitor* OR track* OR model* OR algorithm* OR "machine learning")
• Date when made searches: 2026/01/14
• Limits: none

• Database: ACM
• Search string: [[Abstract: pregnan*] OR [Abstract: perinatal] OR [Abstract: antenatal] OR [Abstract: prenatal] OR [Abstract: postnatal] OR [Abstract: postpartum] OR [Abstract: maternal*]] AND [[Abstract: depression*] OR [Abstract: anxiet*] OR [Abstract: stress*] OR [Abstract: trauma*] OR [Abstract: suicid*] OR [Abstract: self-harm*] OR [Abstract: phobia*] OR [Abstract: emotion*] OR [Abstract: distress] OR [Abstract: mental*] OR [Abstract: psychiatric]] AND [[Abstract: wearable*] OR [Abstract: sensor*] OR [Abstract: smartphone*] OR [Abstract: "mobile phone*"] OR [Abstract: "mobile app*"] OR [Abstract: mhealth] OR [Abstract: smartwatch*] OR [Abstract: "digital phenotyping"] OR [Abstract: "passive sensing"]] AND [[Abstract: predict*] OR [Abstract: forecast*] OR [Abstract: detect*] OR [Abstract: monitor*] OR [Abstract: track*] OR [Abstract: model*] OR [Abstract: algorithm*] OR [Abstract: "machine learning"]]
• Date when made searches: 2026/01/14
• Limits: none

## Supplementary search

• Database: PubMed
• Search string: (pregnan*[tiab] OR perinatal[tiab] OR antenatal[tiab] OR prenatal[tiab] OR postnatal[tiab] OR postpartum[tiab] OR maternal*[tiab]) AND (depression*[tiab] OR anxiet*[tiab] OR stress*[tiab] OR trauma*[tiab] OR suicid*[tiab] OR self-harm*[tiab] OR phobia*[tiab] OR emotion*[tiab] OR distress[tiab] OR mental*[tiab] OR psychiatric[tiab] OR schizophreni*[tiab] OR bipolar*[tiab] OR "eating disorder*"[tiab]) AND (wearable*[tiab] OR sensor*[tiab] OR

smartphone*[tiab] OR "mobile phone*"[tiab] OR "mobile app*"[tiab] OR mhealth[tiab] OR smartwatch*[tiab] OR "digital phenotyping"[tiab] OR "passive sensing"[tiab]) AND (predict*[tiab] OR forecast*[tiab] OR detect*[tiab] OR monitor*[tiab] OR track*[tiab] OR model*[tiab] OR algorithm*[tiab] OR "machine learning"[tiab])
• Date when made searches: 2026/06/22
• Limits: none

• Database: Web of Science
• Search string: (TI=(pregnan* OR perinatal OR antenatal OR prenatal OR postnatal OR postpartum OR maternal*) OR AB=(pregnan* OR perinatal OR antenatal OR prenatal OR postnatal OR postpartum OR maternal*)) AND (TI=(depression* OR anxiet* OR stress* OR trauma* OR suicid* OR self-harm* OR phobia* OR emotion* OR distress OR mental* OR psychiatric OR schizophreni* OR bipolar* OR "eating disorder*") OR AB=(depression* OR anxiet* OR stress* OR trauma* OR suicid* OR self-harm* OR phobia* OR emotion* OR distress OR mental* OR psychiatric OR schizophreni* OR bipolar* OR "eating disorder*")) AND (TI=(wearable* OR sensor* OR smartphone* OR "mobile phone*" OR "mobile app*" OR mhealth OR smartwatch* OR "digital phenotyping" OR "passive sensing") OR AB=(wearable* OR sensor* OR smartphone* OR "mobile phone*" OR "mobile app*" OR mhealth OR smartwatch* OR "digital phenotyping" OR "passive sensing")) AND (TI=(predict* OR forecast* OR detect* OR monitor* OR track* OR model* OR algorithm* OR "machine learning") OR AB=(predict* OR forecast* OR detect* OR monitor* OR track* OR model* OR algorithm* OR "machine learning"))
• Date when made searches: 2026/06/22
• Limits: none

• Database: Scopus
• Search string: TITLE-ABS ( ( pregnan* OR perinatal OR antenatal OR prenatal OR postnatal OR postpartum OR maternal* ) AND ( depression* OR anxiet* OR stress* OR trauma* OR suicid* OR self-harm* OR phobia* OR emotion* OR distress OR mental* OR psychiatric OR schizophreni* OR bipolar* OR "eating disorder*" ) AND ( wearable* OR sensor* OR smartphone* OR "mobile phone*" OR "mobile app*" OR mhealth OR smartwatch* OR "digital phenotyping" OR "passive sensing" ) AND ( predict* OR forecast* OR detect* OR monitor* OR track* OR model* OR algorithm* OR "machine learning" ) )
• Date when made searches: 2026/06/22
• Limits: none

• Database: APA PsycInfo
• Search string: (TI(pregnan* OR perinatal OR antenatal OR prenatal OR postnatal OR postpartum OR maternal*) OR AB(pregnan* OR perinatal OR antenatal OR prenatal OR postnatal OR postpartum OR maternal*)) AND (TI(depression* OR anxiet* OR stress* OR trauma* OR suicid* OR self-harm* OR phobia* OR emotion* OR distress OR mental* OR psychiatric OR schizophreni* OR bipolar* OR "eating disorder*") OR AB(depression* OR anxiet* OR stress* OR trauma* OR suicid* OR self-harm* OR phobia* OR emotion* OR distress OR mental* OR psychiatric OR schizophreni* OR bipolar* OR "eating disorder*")) AND (TI(wearable* OR sensor* OR smartphone* OR "mobile phone*" OR "mobile app*" OR mhealth OR smartwatch* OR "digital phenotyping" OR "passive sensing") OR

AB(wearable* OR sensor* OR smartphone* OR "mobile phone*" OR "mobile app*" OR mhealth OR smartwatch* OR "digital phenotyping" OR "passive sensing")) AND (TI(predict* OR forecast* OR detect* OR monitor* OR track* OR model* OR algorithm* OR "machine learning") OR AB(predict* OR forecast* OR detect* OR monitor* OR track* OR model* OR algorithm* OR "machine learning"))

• Date when made searches: 2026/06/22

• Limits: none

• Database: IEEE Xplore

• Search string:

(pregnan* OR perinatal OR antenatal OR prenatal OR postnatal OR postpartum OR maternal*) AND (depression* OR anxiet* OR stress* OR trauma* OR suicid* OR "self-harm" OR "self harm"OR phobia* OR emotion* OR distress OR mental* OR psychiatric OR schizophreni* OR bipolar OR "eating disorder*") AND (wearable* OR sensor* OR smartphone* OR "mobile phone*" OR "mobile app*" OR mhealth OR smartwatch* OR "digital phenotyping" OR "passive sensing") AND (predict* OR forecast* OR detect* OR monitor* OR track* OR model* OR algorithm* OR "machine learning")

• Date when made searches: 2026/06/22

• Limits: none

• Database: ACM

• Search string:

(Title:(pregnan* OR perinatal OR antenatal OR prenatal OR postnatal OR postpartum OR maternal*) OR Abstract:(pregnan* OR perinatal OR antenatal OR prenatal OR postnatal OR postpartum OR maternal*)) AND (Title:(depression* OR anxiet* OR stress* OR trauma* OR suicid* OR self-harm* OR phobia* OR emotion* OR distress OR mental* OR psychiatric OR schizophreni* OR bipolar* OR "eating disorder*") OR Abstract:(depression* OR anxiet* OR stress* OR trauma* OR suicid* OR self-harm* OR phobia* OR emotion* OR distress OR mental* OR psychiatric OR schizophreni* OR bipolar* OR "eating disorder*")) AND (Title:(wearable* OR sensor* OR smartphone* OR "mobile phone*" OR "mobile app*" OR mhealth OR smartwatch* OR "digital phenotyping" OR "passive sensing") OR Abstract:(wearable* OR sensor* OR smartphone* OR "mobile phone*" OR "mobile app*" OR mhealth OR smartwatch* OR "digital phenotyping" OR "passive sensing")) AND (Title:(predict* OR forecast* OR detect* OR monitor* OR track* OR model* OR algorithm* OR "machine learning") OR Abstract:(predict* OR forecast* OR detect* OR monitor* OR track* OR model* OR algorithm* OR "machine learning"))

• Date when made searches: 2026/06/22

• Limits: none
